# Baking Powder Actuated Centrifugo-Pneumatic Valving for Automation of Multi-Step Bioassays

**DOI:** 10.3390/mi7100175

**Published:** 2016-10-01

**Authors:** David J. Kinahan, Marine Renou, Dirk Kurzbuch, Niamh A. Kilcawley, Éanna Bailey, Macdara T. Glynn, Colette McDonagh, Jens Ducrée

**Affiliations:** 1 School of Physical Sciences, Dublin City University, Glasnevin, Dublin 9, Ireland; renou-m@hotmail.fr (M.R.); dkurzbuch@gmail.com (D.K.); n.kilcawley@gmail.com (N.A.K.); eanna.bailey3@mail.dcu.ie (É.B.); macdarag@gmail.com (M.T.G.); colette.mcdonagh@dcu.ie (C.M.); 2 Biomedical Diagnostics Institute, Dublin City University, Glasnevin, Dublin 9, Ireland; 3 Telecom Physique Strasbourg, Université de Strasbourg, Strasboug CS 10413, France

**Keywords:** Lab-on-a-Disc (LoaD), centrifugal microfluidics, dissolvable film valving

## Abstract

We report a new flow control method for centrifugal microfluidic systems; CO_2_ is released from on-board stored baking powder upon contact with an ancillary liquid. The elevated pressure generated drives the sample into a dead-end pneumatic chamber sealed by a dissolvable film (DF). This liquid incursion wets and dissolves the DF, thus opening the valve. The activation pressure of the DF valve can be tuned by the geometry of the channel upstream of the DF membrane. Through pneumatic coupling with properly dimensioned disc architecture, we established serial cascading of valves, even at a constant spin rate. Similarly, we demonstrate sequential actuation of valves by dividing the disc into a number of distinct pneumatic chambers (separated by DF membranes). Opening these DFs, typically through arrival of a liquid to that location on a disc, permits pressurization of these chambers. This barrier-based scheme provides robust and strictly ordered valve actuation, which is demonstrated by the automation of a multi-step/multi-reagent DNA-based hybridization assay.

## 1. Introduction

Increasingly over the past decade, centrifugal microfluidic systems [1,2,3] have been applied to a variety of application fields such as biomedical diagnostics [4,5,6], bioprocess monitoring [7] and environmental screening [8,9,10]. This “Lab-on-a-Disc” (LoaD) platform is particularly useful for near patient/point-of-care/point-of-use applications, deriving its advantages from the ease with which a sample can be processed without a need for pneumatic interfaces or external pumps. The cartridges have dimensions similar to commonly available optical data storage media such as compact discs^TM^ (CDs). The comparatively simple instrumentation (often just a low-cost spindle motor) and the inherent capability to centrifuge samples such as blood [11,12] are other major benefits of the LoaD platform.

However, as all liquids on-disc are subjected to the same centrifugal field, advanced valving schemes are required to automate a sequences of laboratory unit operations (LUOs) such as mixing, metering and reagent release [13]. On-disc valving can be broadly categorised into three sub-types: externally actuated, rotationally controlled, and event-triggered. 

Externally actuated valves can broadly be defined as those controlled by modules on the peripheral instrument. Such interactions can include provision of external pressure [8], heat to induce phase-changes [14,15,16,17] and physical manipulation [18,19]. While these approaches expand the functionality of the centrifugal platform and also the number of LUOs that can be automated on a single cartridge, they tend to compromise the simplicity of the associated instrument. 

The most common valves are opened by a change of the spin rate and are termed rotationally actuated. Typically, they are based around the interplay between the centrifugally induced hydrostatic pressure and the capillary force acting on liquid in microchannels. Counter pressure induced by compression of entrapped gas might also be used as one of the competing forces. The high-pass version of rotationally actuated valves, which actuate towards elevated spin rates, include capillary burst valves [20,21,22,23,24], dissolvable-film (DF) valves [25], burstable foils [26], elastomeric membranes [27], and dead-end pneumatic chambers [28]. Triggered by a reduction of the spin rate, the low-pass valves include hydrophilic siphons [7,29,30] and pneumatically primed siphons [12,31,32,33,34].

The primary drawback of all rotationally-actuated valves is fidelity and reproducibility of the rotationally induced burst pressures. The burst pressures are intimately linked to the geometry, topography and surface chemistry governing the shape and contact line of the meniscus. Hence, in order to reliably separate subsequent assay steps such as the release of on-disc stored reagents, frequency bands have to be reserved to account for manufacturing tolerances and defects. For rotationally actuated valves, this minimum spacing of burst frequencies tends to severely restrict the number of LUOs that might be implemented in series. 

Many efforts have been made to mitigate this drawback; for example, low-pass and high-pass have been combined [7,29] in series to good effect. In another approach, the release of liquid from a group of valves, triggered simultaneously by a decrease in spin rate, had been staggered through the use of high-resistance microchannels [33].

In a new, recently introduced valving class [35,36], the arrival of liquid in a designated location triggers the subsequent valving steps. In this so-called event-triggered flow control, the layout of the disc-based channel network, rather than changes in disc spin rate, fully determines the order in which valves actuate. Based on (water) dissolvable-film (DF) technology [35,36], these event-triggered valves can function akin to an electrical relay and can enable Boolean-like AND- and OR-conditional triggering [35,36]. 

As event-triggered valves operate essentially independent of the spin rate, the number of assay steps that can be automated on a disc is not restricted by the finite spin rate envelope. However, these valves can result in a relatively large reagent and sample loss due to the dead volume of these valves. Additionally, the intervals between valving steps are prescribed by dissolution of the film, which may notably deviate with reaction or incubation times of the bioassays.

**Figure 1:**
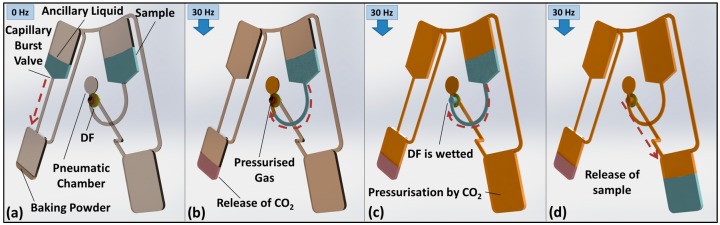
Basic valve operation. Note that the intensity of brown intensity indicates the gas pressure. (**a**) Liquid and baking powder are loaded, and the disc is sealed from the atmosphere using adhesive tape (not shown). In this pneumatically isolated fluidic network, the bypass channels prevent the establishment of uniform pressure. Only the gas pocket at the dissolvable film (DF) membrane is exposed to the hydrostatic pressure head of the upstream sample; (**b**) upon spinning, a capillary burst valve opens and the ancillary liquid reaches the baking powder; the emerging CO_2_ gas pressurises the network and thus compresses the gas pocket; (**c**) with increasing production of CO_2_, the sample protrudes into the pneumatic chamber and eventually wets the DF; and (**d**) DF dissolves to open the valve and release the sample.

In this paper, we present a new mechanism for serial actuation of valves, which is largely independent of the spin rate. The CO_2_ gas issued by household baking powder [37,38] stored on-disc is released by an ancillary liquid to pressurise a sealed microfluidic compartment. Two alternative valve types are demonstrated. In a first approach, which we refer to as “volume governed”, the order of serial actuation of on-disc DF-valves is established through tailoring the volume of their respective pneumatic chambers (Figure 1). 

In the second approach, called “barrier governed”, we demonstrate how the disc can be divided into discrete chambers that are separated by dissolvable films. By sequential dissolution of these DFs, each section of the disc is pressurised in a sequence encoded by the disc architecture, resulting in ordered valve actuation.

We characterise both flow control schemes in terms of their timing. We then present an automated disc, where a sample containing DNA is hybridised with an oligo-spotted substrate and then washed by three buffers.

## 2. Materials and Methods

### 2.1. Disc Assembly, DF Tabs and Experimental Test Stand

The microfluidic discs were assembled using polymer lamination methods. First, layers of 1.5-mm thick Poly(methyl methacrylate) (PMMA) were defined by a laser cutter [39] (Epilog Zing, Golden, CO, USA). Voids in these layers defined loading reservoir features and loading holes/vents. Layers of 86 µm thick pressure sensitive adhesive (PSA, Adhesives Research, Limerick, Ireland) were patterned by a knife-cutter (Graphtec, Yokohama, Japan) to create smaller features, such as microchannels, from voids in PSA.

The disc is manufactured using essentially the same eight-layer configurations described previously [35]. Note that for investigating DNA binding, a Zeonor slide, of identical geometry to a standard microscope slide, was adhered to the base of the disc. This slide was spotted with oligomers and, following completion of experiments, was removed from the disc and measured as described in Section 2.2. The disc is assembled by the following layers:

- Layer 1—PMMA—Loading holes and vents
- Layer 2—PSA—Microchannels
- Layer 3—PMMA—Reservoirs
- Layer 4—PSA—Sealing of DFs
- Layer 5—PSA—Alignment and sealing of DFs
- Layer 6—PMMA—Support for DFs
- Layer 7—PSA—Lower level microchannels
- Layer 8—PMMA—Base
- Layer 9—PSA—For attaching Zeonor slide
- Layer 10—Zeonor—Oligomer-spotted slide

The DF (Barnyarns, Rippon, UK), here termed E-film, is a low-cost material based on polyvinyl alcohol that is commercially available; its primary use is for embroidery. This film has been characterised previously [35] and typically dissolves in the presence of deionized (DI) water in less than 10 s. E-film is non-adhesive, and thus the tabs were created by mounting the film onto double-sided PSA tabs as described previously [25,35].

A specialised experimental test and development instrument known as a spin-stand [40,41], described previously [35], acquires images of the rotating disc. All discs are tested at a spin rate of 30 Hz and accelerated/decelerated at 12.5 Hz·s^−1^.

### 2.2. Binding Assay

Polymer chips were ultrasonically cleaned in 2% surfactant (Micro 90, International Product Corporation, Burlington, NJ, USA) and distilled water, and after drying with nitrogen, activated by oxygen plasma (40 kHz, 100 W, 0.2–1 mbar) for 15 min. The silanization was carried out by immersion in a 3% (*v*/*v*) solution of 3-amino-propyl-triethoxy-silane (APTES) in 95% ethanol for 2 h. The substrates were rinsed with ethanol and water, dried under nitrogen flow, and cured for 1 h in an oven at 80 °C. For immobilization of biomolecules, the surface was mediated by an aldehyde-dextran linker, which was prepared according the protocol in an earlier paper [42].

Amino-functionalized capture probe DNA (5′-NH2-TTCAAAATTGCGAAGTTGGG-3′) was mixed with aldehyde dextran in 3X saline-sodium citrate buffer (SSC, pH 7.0, Sigma Aldrich, St. Louis, MO, USA) and incubated for 30 min at 30 °C. This solution was spotted on the APTES coated slide using a sciFLEXARRAYER S3 (Scienion, Berlin, Germany), a piezo-driven non-contact dispensing system, forming a line of five spots. This was left overnight in a humidity chamber at room temperature. Unbound capture probes were removed by washing them with 2X SSC and sodium dodecyl sulphate (SDS, Sigma-Aldrich), and were then finally dried. 

In addition, 10 µM complimentary DNA (Eurofins, MWG Operon, Ebersberg, Germany) labelled with cy3 (5′-cy3-CCCAACTTCGCAATTTTGAA-3′) was used as the fluorescent target probe. Solutions of varying concentrations of target probe were prepared in 3X SSC, 0.1% SDS and were subsequently loaded into the sample chambers. On-disc, the array was washed by series by three solutions; first, by 2X SSC, 0.2% SDS, secondly with 2X SSC, and finally with 0.2X SSC.

For fluorescence detection, we used the GMS 418 Array Scanner (Genetic MicroSystems Inc., Woburn, MA, USA), which enabled us to measure non-standard sized polymer slides with the dimension of 37.5 cm × 25 cm. We excited the cy3-labelled DNA at a wavelength of 532 nm and measured the fluorescence emission at 570 nm. The images were analysed using ScanArray Express software (Perkin Elmer, Norwalk, CT, USA).

## 3. Valve Concept and Function

### 3.1. Volume-Governed Actuation

Previously, Gorkin et al. [25] introduced valves that were actuated by elevating the spin rate. Similar to the work described here, the DFs were recessed in pneumatic chambers; by displacing the liquid into the chambers, the DFs were dissolved to open a route for the restrained liquid. Oriented with the DFs directly radially outwards of the restrained liquid volume, the burst frequencies of the valves were largely determined by the volume of the pneumatic chamber, the radial location of the valves and the liquid height above the valve. However, the fidelity of these valves is also affected by surface tension that stabilised the dense liquid above the less dense trapped gas.

A further refinement was introduced by Dimov et al. [43], where a radially inward turn in the valve microchannel resulted in the denser liquid being located radially outwards of the less dense gas. This approach attenuated the influence of surface tension and thus improved the predictability of the valves. This design change also engendered a new optimisation parameter—the location of the DF in the pneumatic chamber. However, this improvement was at the expense of increased dead volume in the valves. 

In both approaches, the rotationally induced hydrostatic pressure displaces liquid into the pneumatic chambers until hydrostatic equilibrium has established. In the approach presented here, the additional pressure required to wet the valve is provided by the release of CO_2_ from the baking powder. Note that the volume of baking powder loaded to the disc depends on the density of baking powder and the volume of the chamber; it roughly amounts to ~250 mg. Fordtran et al. [44] have previously studied the gas generation rate of baking soda into finite volumes.

Assuming the liquid volumes and location of the DF within the valves remain unchanged, the pressure required to actuate the valves, *P*_bp_ released from wetted baking powder, follows Boyle’s law

(1) $$P_{\mathrm{bp}}\propto \frac{V_{\mathrm{disp}}}{V_{T}}$$

where *V*_T_ is the entire volume of the pneumatic chamber, and *V*_disp_ represents the additional liquid volume displaced into the valve needed to wet the DF.

**Figure 2:**
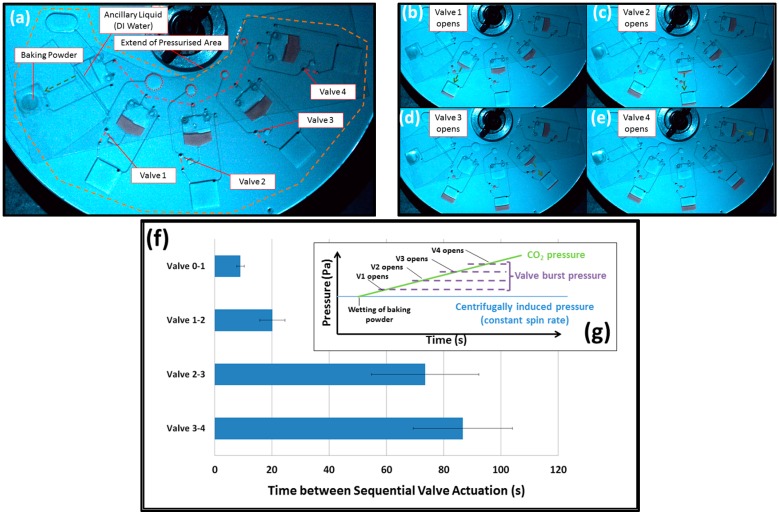
Volume governed valve actuation. (**a**) The baking powder is activated by the ancillary liquid (deionised (DI) water) to pressurize the interconnected cavities on the disc. Pneumatic chambers of each valve are highlighted and **red** dashed lines indicate connecting lower level channels (**b**–**e**). With increasing gas pressure, liquid is pumped into the dead-end pneumatic chambers (valves), successively disintegrating the DF. The order of valve actuation is governed by the compression ratio and thus the size of the pneumatic chambers; Valve 1, the largest chamber, is actuated first; (**f**) timing data of valve actuation (*n* = 6); and (**g**) qualitative representation of the expected gas pressure changes in the disc; the rise in the CO_2_ pressure has the same effect as an increase in spin rate. Note the disc was initially designed so that, in the absence of CO_2_, all DF burst valves will not trigger at or below 30 Hz spin rate.

**Figure 3:**
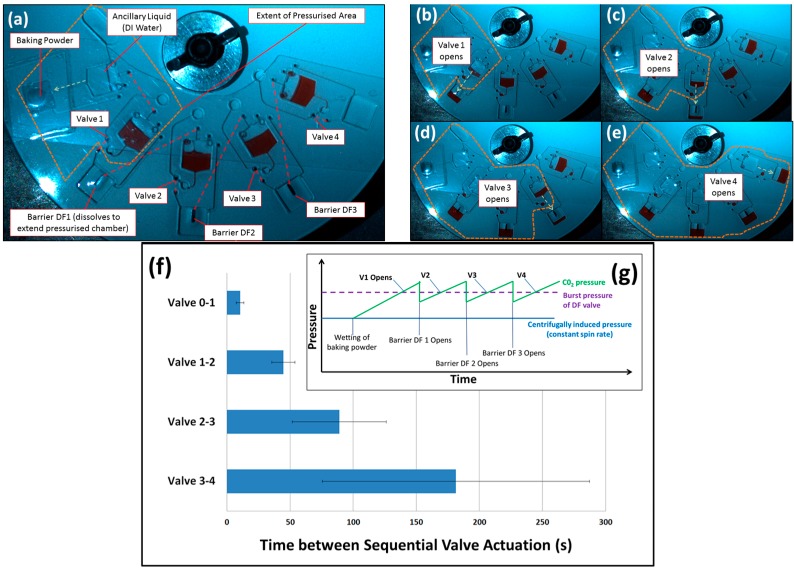
Barrier governed valve actuation. (**a**) Upon contacting the baking powder, the first section of the disc is pressurized to open Valve 1. **Red** dashed lines show lower-level connecting channels; (**b**) the liquid released from Valve 1 dissolves the DF separating the first chamber from the second; this, in turn, exposes Valve 2 to increased gas pressure; (**c**–**e**) the subsequent valves open, and the extent of the pneumatic chamber is shown by the dashed line; and (**f**) time between valve actuations (*n* = 3). Timing begins when the disc starts to spin. In addition, (**g**) is a qualitative representation of the expected gas pressure changes in the disc; the opening of DF barriers between chambers temporarily reduces the average gas pressure across the chambers. Note that the disc was initially designed so that, in the absence of CO_2_, all of DF burst valves will not trigger at or below 30 Hz spin rate.

Figure 2 shows a disc where a single CO_2_ source actuates a series of valves in a well-defined order. In this case, each valve has a different volume *V*_T_. As shown in Figure 3f, the time of valve actuation is governed by *V*_T_; valves with larger pneumatic chambers are triggered before those with smaller pneumatic chambers. While not investigated in this paper, operating the disc at a higher spin rate will reduce *V*_disp_, and so the valves will actuate in a faster sequence.

### 3.2. Barrier-Governed Actuation

In the alternative, barrier-governed actuation, the disc is compartmentalized into separate chambers divided by dissolvable barriers. The gas emerging from the baking powder triggers the first valve. The so-released liquid then wets, and thus removes, the DF to pressurise the subsequent chamber where the next valve is opened.

As shown in Figure 3, this cascading scheme is controlled by liquid movement about the disc, putting in place an event-triggered “hand-shaking” mechanism. However, as the time between valving steps increases with each barrier governed actuation, the volume to be pressurised grows, and thus additional amounts of CO_2_ gas is released.

## 4. Binding Assay

**Figure 4:**
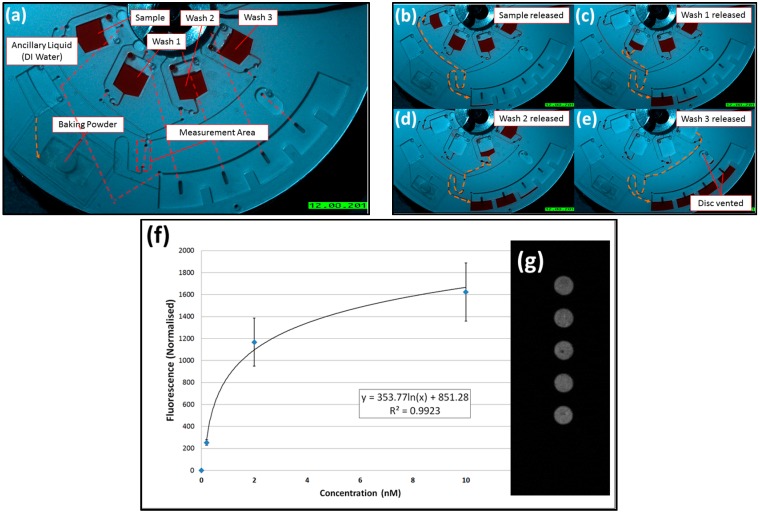
A direct DNA hybridization assay. (**a**–**e**) Visualization of the chip, using food dyes, of the sequential release of sample and washes. Note that the last DF vents the disc to atmosphere. The red dashed lines in (**a**) show lower level connecting channels; (**f**) fluorescence versus loaded sample concentration (*n* = 3 discs); and (**g**) is a representative image showing fluorescent DNA hybridization on the slide. Note the disc was initially designed so that, before the liquid-induced release of CO_2_, the all DF burst valves will not trigger at or below 30 Hz spin rate (see Supplementary Material Video S1).

The barrier-governed valves are adapted to demonstrate on-disc DNA hybridization according to the protocol described in Section 2.2. This disc is shown in Figure 4 with red dyed water for visualisation purposes only.

Immediately prior to testing, the slide is affixed to the bottom side of the disc with the spotted sections aligned with the measurement zone. The disc was then loaded with a 90 µL aliquot of sample, Washes 1–3, baking powder, and deionised (DI) water as ancillary liquid. The disc was then sealed with transparent adhesive tape. 

On increasing the spin rate to 30 Hz, the baking powder is wetted and a compartment encompassing the sample reservoir, washing channels and waste chamber is pressurised. A sample is released through Valve 1, runs over the slide, and is then forwarded into the waste chamber. 

The arrival of the sample into the waste chamber removes a dissolvable barrier, resulting in the pressurisation of the second chamber followed by the release of Wash 1. This sequence continues with Washes 2 and 3 until a final dissolvable barrier is wetted to open a vent (to atmosphere). 

This disc was tested at four different sample concentrations: 0, 0.2, 2, and 10 nM. Results (Figure 4f) show the expected semi-log relation between DNA concentration and measured fluorescence.

## 5. Discussion

We presented a new mechanism for valve actuation through gas release from baking powder that is initially dry-stored on the centrifugal microfluidic disc. The most important advantage of this technology is the option for valving at rather random, e.g., constant spin rate, thus making flow control independent from external instrumentation. Furthermore, for the barrier-governed valves, the order of actuation is strictly defined by the disc architecture. This offers a similar performance to event-triggered valves while allowing simpler disc architecture and reducing dead volume, thus saving precious real estate. While there is some variation in the timing of burst valve actuations, we believe these are a result of manufacturing defects where the valve geometries are not repeatably defined, or, in some cases, where micro-leaks might affect the rate of pressurisation of the discs. Use of an alternative manufacturing method will certainly improve repeatability of the system.

This chemically actuated valving mechanism will increase the exposure of the reagents to CO_2_; this may, in turn, affect performance of certain bioassays. However, in most cases, the relatively short period of point-of-care applications will attenuate this effect. A second challenge is the need to hermetically seal the disc, which might lead to slightly more complex loading procedures. However, this drawback might be relieved through ergonomic design; furthermore, the operation of the disc above atmospheric pressure might, like a positive displacement filter hood, even reduce the risk of assay contamination from the surrounding environment. 

## Supplementary Materials

The following are available online at www.mdpi.com/2072-666X/7/10/175/s1, Video S1: Binding Assay Disc (Dyed Water).
