# Supplementary material for: Baking Powder Actuated Centrifugo-Pneumatic Valving for Automation of Multi-Step Bioassays
Source: Micromachines (Basel). 2016 Oct 1;7(10):175. doi: 10.3390/mi7100175 (PMC6189914; doi:10.3390/mi7100175)
Supplement: Supplementary file 1 [file micromachines-07-00175-s001.zip › Supplementary Material.pdf]

Supplementary Materials: Baking Powder Actuated Centrifugo-Pneumatic Valving for Automation of Multi-Step Bioassays

David J. Kinahan, Marine Renou, Dirk Kurzbuch, Niamh A. Kilcawley, Éanna Bailey, Macdara T. Glynn, Colette McDonagh and Jens Ducrée

1. Calculation of Burst Frequencies

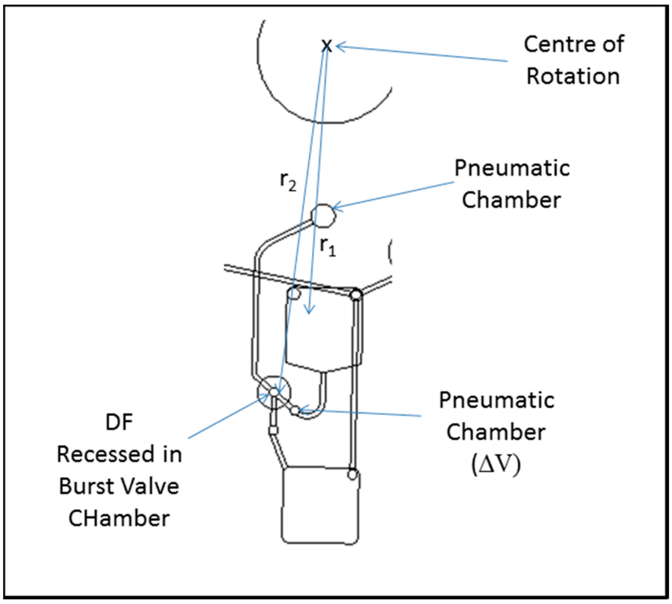

Figure S1. Schematic of valve geometry. Note that this drawing is not to scale.

The disc shown in Figure 2 is composed of four dissolvable film (DF) burst valves that are pressurised simultaneously. In order to burst, liquid must be pushed, via a combination of centrifugal force and the pressurisation caused by wetted baking powder, into a dead end pneumatic chamber. It can be assumed that, at the instant the DF is contacted by water, the valve opens.

The volume of the pneumatic chamber, V_T, is made up of the large radially inwards pneumatic chamber (where each valve has a different size of inward chamber), the pneumatic chamber into which each DF is recessed (1 mm diameter) and the ΔV pneumatic chamber (3.5 mm diameter). The chambers are defined in Poly(methyl methacrylate) (PMMA) of thickness 1.5 mm. However, as this depth is constant, it is assumed to be 1 mm depth. Additionally, microchannels are defined by pressure sensitive adhesive (PSA) of 0.086 mm thickness; they are thus disregarded.

2. Valve Burst Pressure

The burst pressure of each valve is the pressure that must be applied, through a combination of centrifugal force and CO_2 release, to compress the gas in the pneumatic chamber, such that the liquid can contact the DF. Therefore, the gas trapped in the pneumatic chamber must be compressed from $V_1 = V_T$ to $V_2 = V_T - \Delta V$. As it can be assumed, the gas in the pocket was at atmospheric pressure (P_1 = 101,325 Pa), and the burst pressure, P_B can be calculated from Boyles Law:

$$P_1V_1 = P_2V_2$$

Table S1. Burst frequency calculations.

Valve	Dia Burst Chamber	Dia ΔV	Dia Pneumatic Chamber	V_T (μL)	ΔV (μL)	Burst Pressure (Pa)
Valve 1	1 mm	3 mm	5 mm	11.78	2.36	126,656.3
Valve 2	1 mm	3 mm	3 mm	8.63	2.36	139,321.9
Valve 3	1 mm	3 mm	2 mm	7.07	2.36	151,987.5
Valve 4	1 mm	3 mm	1 mm	5.50	2.36	177,318.8

Fully loaded, the inner liquid level in the chambers, r_1, is 27 mm. In order to dissolve the DF, the liquid must reach a point within the pneumatic chamber, r_2, which is 37 mm from the centre of rotation. During testing, the disc is rotated at 30 Hz. Therefore, the maximum possible centrifugally induced hydrostatic pressure that can be generated, with the DFs not bursting, at this spin-rate is:

$$P_h = \omega^2 \varrho \, (r_2^2 - r_1^2) + P_{atm}$$

where ω is the disc rate of rotation in radians, and ϱ is the density of the working fluid. Note that $r_2^2 - r_1^2 = (r_2 + r_1)(r_2 - r_1)/2$, and these terms are commonly written as $r = (r_2 + r_1)/2$ and $\Delta r = (r_2 - r_1)$

Thus, the maximum pressure induced by centrifugation is 124,064 Pa, and this is below the calculated burst frequency for each valve. Thus, none of the valves in this configuration will burst due to centrifugation.

In reality, the back pressure from gas trapped within the valves will cause r_2 to be radially outwards of 37 mm. Similarly, the volume of that the valve was occupied by liquid before bursting will also impact on the radial distance r_1. However, this change in radial distance will be minimal, as the width of the liquid reservoir is significantly larger than the pneumatic chambers. Each valve could be characterised independently to determine the actual locations of the liquid interfaces when the disc is rotated at 30 Hz. However, their irregular geometry makes it unfeasible for this level of analysis.

Substracting the maximum centrifugally induced hydrostatic provides the pressurisation required by the release of CO_2 to open the valves

Table S2. Pressure required from CO_2 release.

Valve	Pressure Required from CO_2 Release (Pa)
Valve 1	2591
Valve 2	15,257
Valve 3	27,922
Valve 4	53,254

Thus, as observed in the experiment, the valves open in sequence as the disc is increasingly pressurised.

Note that this analysis assumes that vacuum effects, caused by liquid pumped via centrifugal force within a sealed disc, are minimal and can be disregarded.

Note that the maximum pressure required is @53 kPa. In the subsequent two discs, the geometry of Valve 2 is adapted to use the 'barrier governed' actuation mechanism. Therefore, each section of the disc must reach ~15 kPa above atmospheric pressure to trigger actuation of the valves.

3. Impact of Mass of Baking Powder Loaded

The following is an approximation, based on the ideal gas law, of the compression that occurs within the discs using the mechanism for triggering the valves.

The following is the reaction that occurs when baking powder is wetted:

$$14\ NaHCO_3 + 5\ Ca(H_2PO_4)_2H_2O \rightarrow 14\ CO_2 + Ca_5(PO_4)_3OH + 7\ Na_2HPO_4 + 18\ H_2O$$

Ignoring the by-products, for every molecule of baking soda in the reaction, a molecule of CO_2 is released. Therefore, in the gas phase:

before reaction*:*

$$P_1V_1 = (n_{air})RT$$

after reaction:

$$P_2V_1 = (n_{air} + n_{CO_2})RT$$

Subtracting these equations:

$$\Delta P = (n_{CO_2})RT/V_1$$

The molar mass of baking soda is ~84 g·mol^{-1}; and the mass of baking powder loaded onto each disc is ~250 mg. Thus, the amount of baking soda on the disc is 0.25/84 = 2.9 × 10^{-3} mol.

The volume of free air on the disc shown in Figure 2 may be estimated as (8 mm × 10 mm × 1.5 mm) × 4 × 2. This is based on eight large chambers of each 120 μL each. This does not account for micro-channels and the volume of the ancillary liquid chamber but may be considered conservative, as it also does not account for the volumes occupied on the discs by the ancillary liquid and dyed water (~480 μL).

Taking the internal volume of free air on the disc as 960 μL, the molar amount of air present is therefore 0.001225 g/cm^3 × 0.96 cm^3/28.97 g/mol = 4.06 × 10^{-5} mol.

Assuming that every molecule of baking soda reacts and releases a molecule of CO_2:

$$\Delta P = n_{CO_2} \times R \times T/V_1$$

$$\Delta P = 2.9 \times 10^{-3} \times 8.314 \times 293/960\ \mu L$$

$$\Delta P = \sim 72.6\ bar$$
